# Potential Effects of Prolonged Water-Only Fasting Followed by a Whole-Plant-Food Diet on Salty and Sweet Taste Sensitivity and Perceived Intensity, Food Liking, and Dietary Intake

**DOI:** 10.7759/cureus.24689

**Published:** 2022-05-03

**Authors:** Toshia R Myers, Bradley Saul, Micaela Karlsen, Andrew Beauchesne, Zrinka Glavas, Mackson Ncube, Ryan Bradley, Alan C Goldhamer

**Affiliations:** 1 Research, TrueNorth Health Foundation, Santa Rosa, USA; 2 Statistics, Target RWE, Durham, USA; 3 Research, American College of Lifestyle Medicine, Chesterfield, USA; 4 Radiology, Tufts University School of Medicine, Boston, USA; 5 Statistics, TrueNorth Health Foundation, Santa Rosa, USA; 6 Research, National University of Natural Medicine, Portland, USA; 7 Nutrition, TrueNorth Health Center, Santa Rosa, USA

**Keywords:** dietary intake, food liking, perceived taste intensity, taste sensitivity, salty taste, sweet taste, whole-plant-food diet, prolonged water-only fasting

## Abstract

The overconsumption of calorie-dense foods high in added salt, sugar, and fat is a major contributor to current rates of obesity, and methods to reduce consumption are needed. Prolonged water-only fasting followed by an exclusively whole-plant-food diet free of added salt, oil, and sugar may reduce the consumption of these hyper-palatable foods, but such effects have not been quantified. Therefore, we conducted a preliminary study to estimate the effects of this intervention on salty and sweet taste detection and recognition thresholds and perceived taste intensity after at least five days of fasting and at refeed day three. We also assessed the effects on sweet, salty, and fatty food preference and overall dietary consumption 30 days after the day three refeed visit. Based on this data, we estimated that 10 days after the start of the fasting, salty taste recognition, sweet taste detection, and sweet taste recognition thresholds decreased significantly, salty taste intensity ratings increased significantly, and sweet taste intensity ratings decreased significantly. We also have preliminary data that prolonged water-only fasting followed by refeeding on an exclusively whole-food-plant diet may reduce salty/fatty and sweet/fatty food liking, reduce sugar intake, and increase vegetable intake. These results support further research into the effects of fasting and diet on taste function and food likability and consumption.

## Introduction

Global obesity has more than tripled in the last 30 years, affecting more than 70 million adults and 13 million children in the United States alone. Obesity is correlated with all-cause mortality and is among the leading preventable causes of death worldwide [1]. One explanation for rising obesity rates is the overconsumption of calorie-dense, nutrient-poor foods high in salt, sugar, and/or fat [2]. Sugar, salty, and fatty foods are also hyper-palatable [3], which may contribute to their overconsumption [4].

Human taste sensation begins when molecules in food and drink bind to taste bud receptors on the tongue (e.g., sucrose binds to the heterodimer T1R2-T1R3) [5], activating downstream neurological processes enabling humans to distinguish sweet, salty, sour, bitter, and umami tastes as well as sense and respond to nutrient intake [2]. Data regarding the effects of taste sensitivity, perceived taste intensity, and/or taste liking on food consumption or vice versa is largely inconclusive but studies suggest that taste may be malleable in response to dietary change [6]. Data also suggest that palatability may be a better indicator of food consumption than taste sensitivity or perceived intensity [7].

Prolonged water-only fasting, defined as the complete abstinence of food and liquids except for pure water for >48 hours, is an established, well-tolerated method of therapeutic fasting that has been practiced for millennia to treat a variety of conditions and has been found to be safe under medical supervision [8]. Clinical observation led to the hypothesis that prolonged water-only fasting followed by an exclusively whole-plant-food diet results in decreased salty and sweet food palatability along with reduced obesogenic food and increased whole-plant food consumption [9]. As an initial step in testing this hypothesis, we conducted a preliminary study to estimate the effects of the intervention on salty and sweet taste sensitivity and perceived taste intensity as well as the longer term effects on food preference and dietary consumption.

## Materials and methods

Ethics statement

This study was approved by the Institutional Review Board at the National University for Natural Medicine (RB61917, 10/2017). The research was conducted as described in the approved protocol and complied with the standards of the Declaration of Helsinki.

Study participants

Participants in this study were recruited from incoming patients at the TrueNorth Health Center. Between October 2017 and November 2018, we enrolled 30 consenting participants ages 18-75 years who were elected and were approved by a non-research clinician to undergo a water-only fast lasting five to 14 days. Patients were ineligible if they had been to the health center or smoked tobacco or marijuana products within the last 12 months, ate a diet free of added salt, oil, or sugar, had a self-reported taste or smell disorder, had active cancer, diabetes, or hypertension diagnosis, were pregnant or lactating within the last three months, or had a known allergy to potential testing compounds (sodium chloride, sucrose, citric acid, or caffeine). One of 30 participants did not complete the dietary screener questionnaire (DSQ) for unknown reasons. Six of 30 participants were unable to complete the study requirements due to a change in treatment plan length unrelated to fasting. One out of 30 participants withdrew from the study due to nausea. Of participants completing the refeed day 3 visit (n=23), 20 completed the web-based DSQ and 13 completed the web-based food-liking questionnaire at the remote, follow-up visit.

Study protocol and data management

Enrolled participants attended study visits at baseline (before the start of fasting), after five days of fasting (FD5), the final day of fasting in participants fasting between 10 and 14 days, and after three days of refeeding (RD3) on an exclusively whole-plant-food diet free of added salt, oil, and sugar as previously described [8]. At each visit, participants had weight and blood pressure measured and completed taste detection and recognition threshold (DT and RT) and intensity rating assays as described below. At baseline, participants also reported demographics, had their height measured, and completed the DSQ and food-liking questionnaire (FLQ) as described below. At a remote, follow-up visit 30-days after RD3, participants completed the same independently accessed, web-based DSQ and FLQ. Study data were collected, stored, and managed using REDCap electronic data capture tools hosted at the TrueNorth Health Foundation.

Water-only fasting and refeeding

Water-only fasting and refeeding on an exclusively whole-plant-food diet free of added salt, oil, and sugar were medically supervised by an independent clinician specializing in water-only fasting and refeeding at the TrueNorth Health Center (TNHC). Research personnel had no input into the participants’ treatment plans. The water-only fasting protocol reportedly used at TNHC has been previously described and found to be tolerable and low risk [8].

Salty and sweet taste stimuli

For DT and RT assays, sodium chloride (2.7, 4.1, 5.8, 8.2, 11.8, 16.8, 24.0, 34.2, 68.4, and 137mM) and sucrose 1.0, 1.6, 2.7, 4.5, 7.5, 12.6, 21.0, 35.0, 70, and 140mM were prepared in 10 concentrations in accordance with the International Standards Organization (ISO 2011) as previously described [10,11]. For suprathreshold assays, four (zero, low, medium, high) concentrations of sucrose (0mM, 100mM, 200mM, 400mM) and sodium chloride (0mM, 50mM, 100mM, 200mM) were used [10,11]. Sodium chloride and sucrose were dissolved in distilled water and stored at 4^◦^C. Each sample was given a unique five-digit blinding code as previously described [12].

DT and RT assays

DT is the lowest measurable concentration that a stimulus is detected as something other than water. RT is the lowest measurable concentration that a stimulus is recognized as having the correct characteristic taste (e.g., sodium chloride is salty and sucrose is sweet). DT and RT assays were conducted in a private room that minimized outside auditory, olfactory, and visual stimuli. Participants were instructed to refrain from eating, drinking (except for room temperature, distilled water), brushing teeth, and chewing gum up to one hour prior to testing. Test administrators and participants were blinded to testing solutions.

A standard three-alternative-forced-choice (3AFC) method was used to determine sweet and salty taste DT and RTs as previously described [10-12]. Briefly, the assay consisted of presenting participants with three 10mL samples labeled with a five-digit numbering system. The first group contained three samples of distilled water and the following groups contained two samples of distilled water and one sample of the test stimuli in ascending concentration. Sample order within groups was randomized. For each trial, participants were asked to identify which of the three samples differed from the other two and whether or not they recognized the stimulus as sweet, salty, sour, bitter, or umami. There was not an option for participants to indicate that they could not tell a difference between samples, thus they were required to indicate a sample that was different from the others. Participants were instructed to follow the sip-and-spit procedure, in which they swish a solution for five seconds, spit it out, record results, and then rinse with distilled water. Trials were separated by at least 15 seconds.

Suprathreshold intensity rating assay

Intensity ratings are commonly used to assess perceived taste intensity [10]. Assays were conducted in the same room and directly following the DT and RT assays. Participants were similarly asked to refrain from eating, drinking (except for room temperature, distilled water), brushing teeth, and chewing gum up to one hour prior to testing. Test administrators and participants were both blinded to testing solutions. Perceived intensity was assessed using four different concentrations of sodium chloride and sucrose [10]. For each stimuli, the samples were presented randomly. Participants followed the same swish-and-spit procedure and then rated the intensity of each concentration using a visual analog scale (VAS) graded from 0 to 100 where 0 = no detectable taste and 100 = extremely intense taste as previously described [13].

Food-liking and DSQs

Participants’ preference for standard foods was assessed using a modified version of the PrefQuest food liking questionnaire (see Appendix Table 6 for full questionnaire) [14]. The questionnaire was translated from French to English and French foods uncommonly eaten in the USA were exchanged for comparable American foods. The modified version was beta tested on 40 people and adapted further to correct for participant feedback. Participants’ dietary consumption was assessed using the web-based, self-administered DSQ from the National Health and Nutrition Examination Survey (NHANES) 2009-2010 series as described [15].

Statistical analysis

The sample size for this study was determined on a model-based simulation study of changes in suprathreshold intensity from day 0 to day 5 of fasting. The target of the simulation was estimating 90% confidence intervals (CI) instead of power. This approach for justifying pilot study sample size is recommended when little is known about the parameter(s) of interest over a hypothesis testing approach [16]. Based on having no knowledge of fasting effects on taste adaptation and limited knowledge of taste adaptation in general, we judge that a width of 1.5 or less is an acceptable precision for the range of plausible values (i.e., confidence intervals) for each effect size. Based on the simulation study, a sample size of 20 appears sufficient to create 90% confidence intervals with a width of less than 1.5 for each taste. To accommodate possible loss to follow-up, we recruited a sample size of 30 for this pilot study. The scripts and reports used to obtain sample size estimates are available upon request.

Descriptive statistics were calculated for demographics, clinical characteristics, fasting lengths, as well as mean health metrics at baseline, fasting day 5, and refeed day 3. DSQ data were analyzed to calculate predicted intakes of fiber (g), whole grains (oz equivalents), total added sugars (tsp equivalents), added sugars from sugar-sweetened beverages (SSBs) (tsp equivalents), vegetables (cup equivalents), fruit (cup equivalents) at baseline and 30-day post-RD3 visits. Two-tailed, paired t-tests were used to estimate mean differences in health metrics comparing RD3 vs. baseline and predicted intakes of foods and nutrients comparing 30 days after RD3 vs. baseline. DT and RT were summarized by mean and standard deviations for each assay and time point; moreover, generalized estimating equations (GEEs) were fit to estimate changes in DT and RT 10 days after starting water-only fasting. For analysis of perceived intensity ratings, we excluded the zero concentrations as only five of the 160 tests had a value other than zero at this concentration. The mean and standard deviation of perceived intensity responses were computed for each assay, concentration, and time point. GEEs were fit to estimate intensity ratings 10 days after starting the water-only fast. Note that 10 days after starting water-only fasting, some participants were still in the fasting stage, some were in the refeeding stage, and some had completed refeeding.

To estimate the marginal effect of days since start of fast on intensity ratings, DTs, and RTs, GEE [17] models with log(1+response) were fit with terms for days since start of fast, log10(concentration), and their interaction. Models were fit separately within each taste. Parameter estimates on the original scale and point-wise 90% CIs were obtained by the Delta method. For food liking and diet consumption, two tailed paired t-tests were used to estimate mean differences comparing survey responses collected at baseline to survey responses collected remotely 30 days after departure. SAS 9.4 (Cary, NC) was used to analyze demographic, health metric, and food and nutrient data. Taste DT and RT, suprathreshold, and food-liking data analyses were done in R version 3.6.1 (2019-07-05) [18] using the geepack [19], geex [20], and ggplot2 [21] packages. The R scripts used to run these models are available upon request.

## Results

Participant demographics, clinical, and visit characteristics

Of the 30 participants enrolled at baseline, 25 completed taste assays at FD5 of which 23 completed taste assays at RD3. Of the remaining 23 participants, 20 and 13 completed the follow-up DSQ and FLQ, respectively. At baseline (n=30), 67% of participants were female and the mean (SD) age and BMI were 47.9 (12.2) years old and 29.6 (8.2) kg/m^2^, respectively (Tables 1, 2). There were no clinically meaningful differences between the baseline characteristics of the 30 participants initially enrolled, the 25 participants remaining at FD5, and the 23 participants remaining at RD3 (Appendix Table 7). We observed reductions in weight (-5.4 kg; P<.001), body mass index (BMI) (-1.8 kg/m2; P<.001), and systolic blood pressure (SBP) (-4.5 mmHg; P=.04) at RD3 compared to baseline (n=23; Table 2). There was no significant change observed in diastolic blood pressure (DBP) (-0.23 mmHg; P=.89) (Table 2). The mean fasting length (range) was nine (5, 13) days (Table 1).

**Table 1:** Baseline demographic and visit characteristics SD, standard deviation; n=30.

Characteristics	
Age in years (mean [SD])	47.44 (13.30)
Percent Female (n=20)	66.7
Days Fasted (mean [range])	9 (5, 13)

Salty and sweet taste DT and RT

To assess the effect of water-only fasting and refeeding on salty and sweet taste sensitivity, we used the 3AFC method to measure mean differences in DT and RT of 10 different concentrations of sodium chloride and sucrose, respectively (see Materials and Methods). Mean DT concentrations for sodium chloride remained unchanged at FD5 (4.6mM) and at RD3 (4.2mM) compared to baseline (4.3mM; Table 3). The mean sodium chloride concentrations at which salty taste was recognized changed from 40.8mM at baseline to 31.0mM at FD5 and 26.4mM at RD3 (Table 3). Mean DT concentrations for sucrose were also unchanged at FD5 (15.2mM) and RD3 (8.1mM) compared to baseline (11.6mM; Table 3). The mean sucrose concentrations at which sweet taste was recognized were unchanged from 25.7mM at baseline to 18.2mM at FD5 and 18.2mM at RD3 (Table 3).

**Table 2:** Salty and sweet taste detection and recognition thresholds Values presented as mean (standard deviation). NaCl, sodium chloride; DT, detection threshold; RT, recognition threshold; mM, millimolar; n=23.

Tastant	Baseline	Fast Day 5	Refeed Day 3
NaCl DT	4.3mM (2.12)	4.6mM (3.06)	4.2mM (2.25)
NaCl RT	40.8mM (46.24)	31.0mM (32.15)	26.4mM (38.68)
Sucrose DT	11.6mM (7.38)	15.2mM (20.92)	8.1mM (7.10)
Sucrose RT	25.7mM (25.96)	18.2mM (16.51)	18.2mM (28.50)

**Figure 1:**
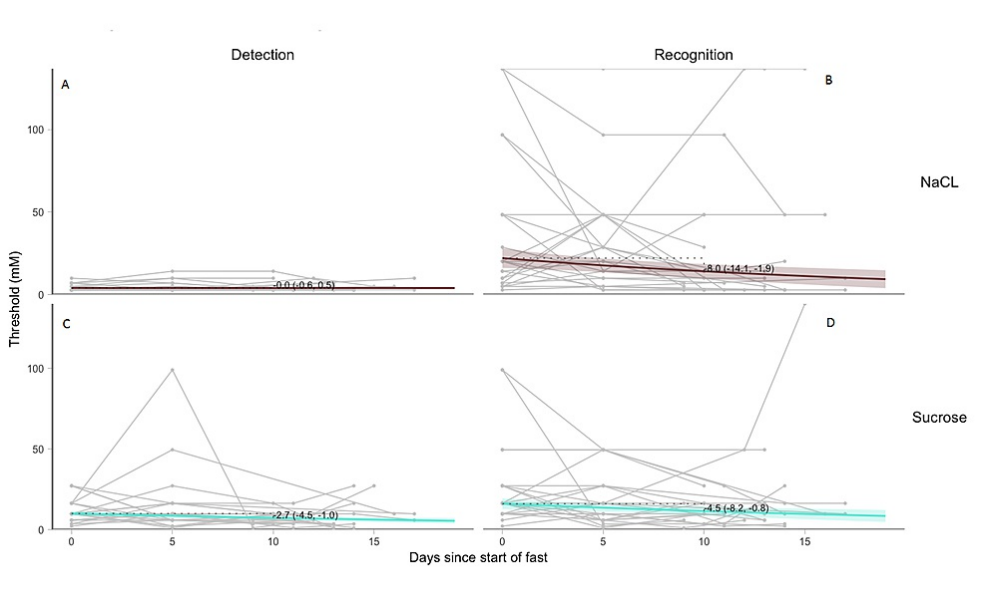
Changes in salty (NaCl) and sweet (sucrose) detection and recognition thresholds overtime Actual (grey) and estimated (blue) NaCl detection (A) and recognition (B) and sucrose detection (C) and recognition (D) thresholds from days since start of fast. Each grey line is a single participant’s trajectory. Colored lines show GEE model fit with pointwise 90% confidence intervals. Value is estimated change 10 days from start of fast with 90% confidence intervals. NaCl, sodium chloride; mM, millimolar.

GEE models were fit to estimate changes in threshold concentrations 10 days after starting water-only fasting. The GEE model estimates that 10 days after starting water-only fasting the mean (90% CI) salty taste DT and RT concentrations would decrease by -0.0mM (-0.6, 0.5) and -8.0mM (-14.1, -1.9), respectively. The GEE model estimates that 10 days after starting water-only fasting the mean (90% CI) sweet taste DT and RT concentrations would decrease by -2.7mM (-4.5, -1.0) and -4.5mM (-8.2, -0.8), respectively (Figures 1A-1D).

Salty and sweet perceived intensity ratings

We assessed the effects of water-only fasting and refeeding on perceived salty and sweet taste suprathreshold intensities by comparing mean VAS ratings (0-100) for four concentrations of sodium chloride and sucrose, respectively. Mean salty taste VAS intensity ratings uniformly increased towards more intense at FD5 and RD3 compared to baseline for all non-zero sodium chloride concentrations (Table 4). For example, at a sodium chloride concentration of 50mM, mean (SD) salty taste intensity ratings increased from 24.7 (14.21) points at baseline to 30.5 (23.94) points at FD5 and 34.8 (22.23) points at RD3 (Table 4). Mean sweet taste VAS intensity ratings remained unchanged or decreased slightly towards less intense at FD5 and/or RD3 compared to baseline (Table 4). For example, at a sucrose concentration of 100mM, mean (SD) sweet taste intensity decreased from 30.0 (13.14) points at baseline to 29.3 (17.01) points at FD5 (Table 4) and 24.5 (10.80) at RD3.

**Table 3:** Salty and Sweet Suprathreshold VAS Intensity Ratings Values are presented as mean (standard deviation). VAS; visual analog scale; mM, millimolar; NaCl, sodium chloride; n=23. VAS from 0 (not detectable taste) to 100 (extremely intense taste).

Tastant (mM)	Baseline	Fast Day 5	Refeed Day 3
NaCl (0)	0.4 (2.09)	0.4 (2.09)	0.0 (0.21)
NaCl (50)	24.7 (14.21)	30.5 (23.94)	34.8 (22.23)
NaCl (100)	56.9 (16.64)	63.7 (20.90)	62.6 (22.88)
NaCl (200)	75.7 (17.08)	78.3 (20.57)	79.5 (21.51)
Sucrose (0)	0.9 (4.17)	0.0 (0.00)	2.2 (10.43)
Sucrose (100)	30.0 (13.14)	29.3 (17.01)	24.5 (10.80)
Sucrose (200)	55.8 (15.42)	59.8 (24.30)	49.3 (20.24)
Sucrose (400)	78.3 (13.78)	75.6 (20.53)	77.7 (16.04)

**Figure 2:**
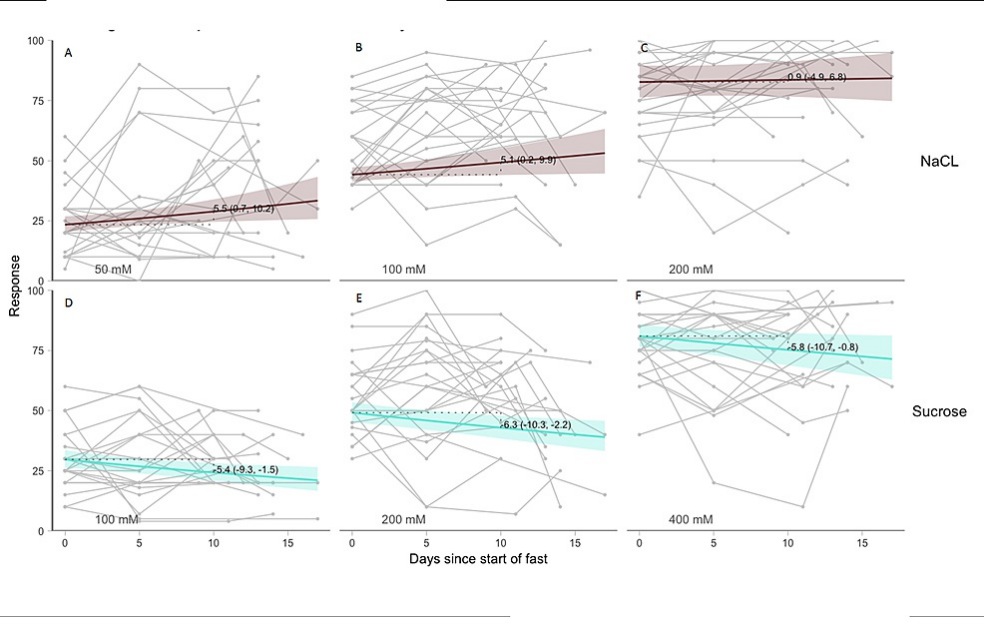
Changes in salty (NaCl) and sweet (sucrose) suprathreshold VAS responses for each concentration overtime VAS ratings for NaCl at concentrations 50mM (A), 100mM (B), and 200mM (C) and sucrose at concentrations 100mM (D), 200mM (E), and 400mM (F). VAS ratings from 0-100 where 0 = no detectable taste and 100 = extremely intense taste. Each line is a single participant’s trajectory. Colored lines show GEE model fit with pointwise 90% confidence intervals. Value is estimated change from baseline to fast day 10 with 90% confidence intervals. NaCl, sodium chloride; mM, millimolar.

GEE models were fit to estimate changes in perceived salty and sweet taste intensity ratings overtime. The GEE model estimates that 10 days after starting water-only fasting the mean (90% CI) salty taste VAS intensity rating would increase by 5.5 (0.7, 10.2), 5.1 (0.2, 9.9), and 0.9 (-4.9, 6.8) points for the 50, 100, and 200mM sodium chloride concentrations, respectively. The GEE model estimates that 10 days after starting of water-only fasting the mean (90% CI) sweet taste VAS intensity rating would decrease by -5.4 (-9.3, -1.5), -6.3 (-10.3, -2.2), and -5.8 (-10.7, -0.8) points for the 100, 200, and 400mM sucrose concentrations, respectively (Figures 2A-2F).

Food liking and diet consumption

**Figure 3:**
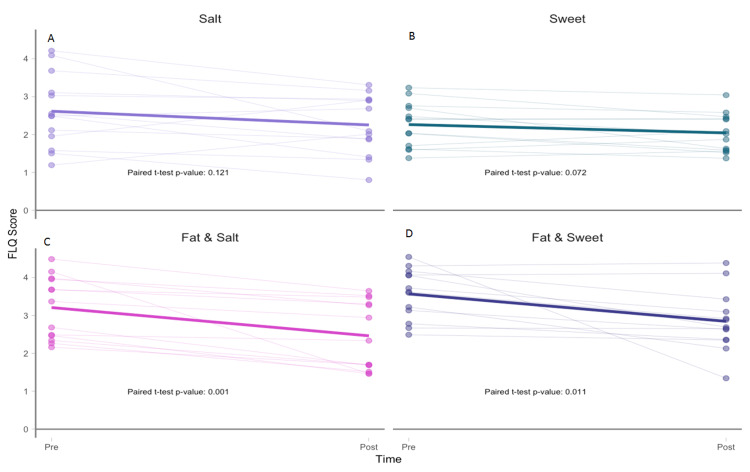
Changes in salty, sweet, fatty and salty, and fatty and sweet food liking between baseline and the 30-day follow-up visit Participant (individual dots) ratings for salty (A), sweet (B), salty and fatty (C), and sweet and fatty (D) foods at baseline and 30 days after RD3. Scale is from zero (dislike) to four (like a lot). Solid line is mean. n=13.

We assessed changes in food liking using a previously validated salty, sweet, and fatty food-liking questionnaire (FLQ) that was adapted to American English and administered on-site at baseline and remotely 30 days after RD3. Food liking is calculated on a scale of 1 to 4 with a higher number indicating increased liking. Of the 23 participants who completed the RD3 visit, only 13 completed the follow-up FLQ. Figures 3A-3D show that for salty taste the FLQ score dropped by -0.36 points (P=.121), for sweet taste the FLQ score dropped by -0.22 points (P=.072), for fatty and salty tastes the FLQ score dropped by -0.74 points (P=.001), and for fatty and sweet tastes the FLQ score dropped by -0.72 points (P=.011).

**Figure 4:**
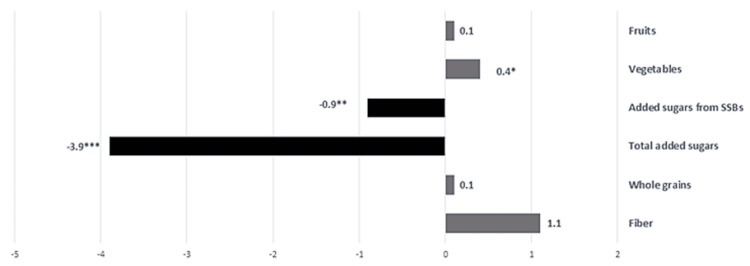
Changes in DSQ predicted food and nutrient consumption between baseline and 30-day follow-up visits DSQ, Dietary Screener Questionnaire; SSB, sugar sweetened beverages; fruits = cup equivalents; vegetables = cup equivalents; added sugars and added sugars from SSBs = tsp equivalents; whole grains = oz equivalents; fiber = g. *P<.05, **P<.001, ***P<.0001; n=20.

We assessed changes in food consumption using the NHANES web-based DSQ that was administered onsite at baseline and remotely 30 days after RD3. Out of the 23 participants who completed the RD3 visit, 20 completed the DSQ at the follow-up visit. The DSQ measures food consumption over the 30 previous days. Compared to baseline, at the 30-day, follow-up visit, there was a significant increase in cups of vegetables (0.4; P=.0054) and a significant decrease in the grams of total added sugar (-3.9; P<.0001) and added sugar from sugar sweetened beverages consumed (-0.9; P=.0084) (Table 5 and Figure 4).

**Table 4:** Differences in predicted intakes of foods and nutrients based on Dietary Screener Questionnaire ^1^Vegetables including legumes and excluding French fries. ^#^29 participants completed the DSQ at baseline and 20 completed the DSQ at the follow-up visit. g, gram; oz, ounce; tsp, teaspoon;

Predicted intake per day of:	Baseline (N=29)^#^	Follow-up (n=20)	Mean Difference (p-value) (n=20)
Fiber (g)	20.8 (4.2)	21.7 (4.0)	1.1 (0.17)
Whole grains (oz equivalents)	0.9 (0.4)	0.83 (0.3)	0.1 (0.50)
Total added sugars (tsp equivalents)	14.4 (4.0)	10.6 (1.0)	-3.8 (<0.001)
Added sugars from sugar-sweetened beverages (tsp equivalents)	5.1 (1.9)	4.0 (0.5)	-0.9 (0.0084)
Vegetables^1^ (cup equivalents)	2.2 (0.7)	2.5 (0.6)	0.3 (0.0054)
Fruits (cup equivalents)	1.4 (0.7)	1.5 (0.8)	0.1 (0.38)

## Discussion

The complex interrelationship between human taste and nutrient intake is poorly understood. It is especially hard to draw conclusions because of heterogeneous research methodologies and a lack of knowledge regarding the clinical meaningfulness of taste function measures. Nevertheless, increasingly high rates of obesity and chronic disease necessitate the need for continued research into interventions that may naturally reduce obesogenic food consumption. To this end, we conducted the first observational study measuring the effects of water-only fasting followed by a whole-plant-food diet on salty and sweet taste function. These data were then used in GEE modeling to estimate effects 10 days after starting water-only fasting followed by an exclusively whole-plant-food diet. Food liking and intake were also assessed 30 days after RD3.

We observed a trend toward decreased mean sodium chloride and sucrose DT and RT concentrations after five days of water-only fasting that was further supported by GEE modeling estimates. This is in agreement with previous data showing that salty and sweet taste recognition sensitivity increased after only 13 hours of water-only fasting [22]. It is accepted that individuals with lower DT and RT have increased taste sensitivity [10], but standard threshold ranges, if/how one threshold affects the other, and if/how they are correlated to taste preference and/or dietary consumption (let alone health outcomes) has not been elucidated. Therefore, it is difficult to draw conclusions beyond that fasting and diet may modulate salty and sweet taste function toward increased sensitivity.

Perceived taste intensity represents the perceived concentration of a taste that is above the RT [10]. GEE models estimated that there was a uniform trend toward increased perceived salty taste intensity. Conversely, GEE models estimated that there was a uniform trend toward decreased perceived sweet taste intensity. Given the relatively small change in perceived salty and sweet taste intensities, these changes may not be biologically or clinically meaningful. However, previous studies have found that decreasing salt and sugar consumption reduced their respective perceived intensities [6,23], and perceived sucrose taste intensity (as well as palatability) may be a factor in overconsumption of sweet foods and beverages [24].

Indeed, we observed a trend toward decreased salty and sweet food liking and a significant decrease in salty and fatty and sweet and fatty food liking at the follow-up visit. Additionally, there was a decrease in predicted sugar and sugary beverage intake and an increase in vegetable and fiber intake at the follow-up visit. The DSQ does not report specifically on salt or fat intake, so we are unable to assess if salty (or fatty) food consumption also declined, but the data suggest vegetable consumption increased. Nonetheless, these preliminary results provide support for the hypothesis that water-only fasting followed by a whole-plant-food diet may reduce preference for salty, sweet, and fatty foods and alter dietary consumption accordingly.

Salty taste mechanisms occur through both taste buds and trigeminal fibers and remain poorly defined relative to other tastes. Interestingly, oral microbiota composition was found to impact taste function with an increased abundance of six specific taxa associated with lower salty taste thresholds [25]. It is unknown if fasting affects oral microbiota but there is evidence that a “vegan” diet affects oral microbiota diversity, structure, and relative abundance [26]. Natriuresis is a well-known consequence of fasting, particularly in obese or hypertensive individuals [27], but this effect is unlikely to be related to the increased salty taste sensitivity and perceived intensity observed after fasting as there is evidence that sodium balance is not regulated by taste function [28].

Sweet taste mechanisms are fairly well defined and appear to function differently than other tastes in that there are sweet taste receptors throughout the body, including on cells in the gastrointestinal tract and central nervous system [5]. Additionally, sweet taste sensitivity, but not salty, sour, bitter, or umami, varies throughout the day with lower RTs observed in the morning and higher RTs at night in a pattern that is positively correlated with leptin levels and negatively correlated with blood glucose levels [29]. Fasting also decreases leptin and blood glucose levels [30], which may be one potential way in which fasting affects sweet taste sensation and perception.

This study has notable limitations including that participant dropout successively increased over the course of the study with only 46% of participants completing the FLQ at the follow-up visit. This combined with the low sample size at baseline and high inter-individual variations in taste may have affected our ability to detect statistically significant changes in threshold concentrations. As such, future studies should utilize a priori sample size calculations based on the effect sizes found in our study and employ strategies to improve retention rates and refine inclusion-exclusion criteria to minimize population variability. Additionally, the FLQ, which was translated from French to English, has not been formally tested for reliability and validity and an independent study is needed. The questionnaire also consisted of over 80 questions, which may account for the low recidivism in this questionnaire. The DSQ also did not provide information on salt or fat consumption, which prevented the assessment of the effects of the intervention on the consumption of these foods. Therefore, it may be useful to use other/additional methods or questionnaires to assess taste function as well as food liking and dietary consumption in future studies.

## Conclusions

Obesity is a global issue that undoubtedly stems from unprecedented access to hyper-palatable foods with unnatural amounts of salt, sugar, and/or fat. That taste plasticity may be altered to affect palatability in such a way as to reduce the preference for and consumption of these foods (and promote the consumption of healthy foods) is appealing. This study began a preliminary assessment into the effects of prolonged water-only fasting and refeeding with an exclusively whole-plant-food diet free of added salt, oil, and sugar on changes in taste function as well as food liking and consumption. Overall, the data are encouraging but additional research is necessary to determine if prolonged water-only fasting and refeeding may increase salty and sweet taste sensitivity, decrease liking of sweet, salty, and fatty foods, decrease sugar consumption, and increase vegetable consumption. Future studies continuing this work may benefit by measuring additional tastes, especially fatty, as well as by focusing on taste hedonics using foods with a complete taste profile rather than isolated tastants.

## Appendices

**Table 5:** Food Liking Questionnaire

Food Liking Questionnaire : This questionnaire is used to determine your food preferences. It is composed of 4 parts: Part I: Questions to determine how much you like various foods. Part II: Questions to determine how you prefer to eat various foods. Part III: Questions to determine your favorite beverages. Part IV: Questions to determine your food consumption behavior. Please answer all questions spontaneously. The answers should reflect your preferences and not necessarily how you currently eat. There are no right or wrong answers.			
Part 1 Instructions : The objective is to measure how much you like various foods. For each food item presented, use the scale provided to indicate how much you like it. For example, "How much do you like baked chicken?" If you are indifferent to baked chicken (you neither like nor dislike it), then check the box in the middle of the scale. If you have never tasted the food item, check the box "Never Tasted". Please answer spontaneously. There are no right or wrong answers.			
p1_q1	How much do you like salty snacks (e.g., pretzels, crackers, biscuits, etc.)?		0 Never Tasted
			1 Dislike Extremely
			2 Dislike Very Much
			3 Dislike Moderately
			4 Dislike Slightly
			5 Neither Like nor Dislike
			6 Like Slightly
			7 Like Moderately
			8 Like Very Much
			9 Like Extremely
p1_q2		How much do you like potato chips?	0 Never Tasted
			1 Dislike Extremely
			2 Dislike Very Much
			3 Dislike Moderately
			4 Dislike Slightly
			5 Neither Like nor Dislike
			6 Like Slightly
			7 Like Moderately
			8 Like Very Much
			9 Like Extremely
p1_q3		How much do you like salted peanuts?	0 Never Tasted
			1 Dislike Extremely
			2 Dislike Very Much
			3 Dislike Moderately
			4 Dislike Slightly
			5 Neither Like nor Dislike
			6 Like Slightly
			7 Like Moderately
			8 Like Very Much
			9 Like Extremely
p1_q4		How much do you like breakfast sausage (pork, beef, chicken, turkey, etc.)?	0 Never Tasted
			1 Dislike Extremely
			2 Dislike Very Much
			3 Dislike Moderately
			4 Dislike Slightly
			5 Neither Like nor Dislike
			6 Like Slightly
			7 Like Moderately
			8 Like Very Much
			9 Like Extremely
p1_q5		How much do you like smoked ham?	0 Never Tasted
			1 Dislike Extremely
			2 Dislike Very Much
			3 Dislike Moderately
			4 Dislike Slightly
			5 Neither Like nor Dislike
			6 Like Slightly
			7 Like Moderately
			8 Like Very Much
			9 Like Extremely
p1_q6		How much do you like bratwurst (veal, pork, beef, etc.)?	0 Never Tasted
			1 Dislike Extremely
			2 Dislike Very Much
			3 Dislike Moderately
			4 Dislike Slightly
			5 Neither Like nor Dislike
			6 Like Slightly
			7 Like Moderately
			8 Like Very Much
			9 Like Extremely
p1_q7		How much do you like deli meats (i.e., roast beef, bologna, ham, turkey, etc.)?	0 Never Tasted
			1 Dislike Extremely
			2 Dislike Very Much
			3 Dislike Moderately
			4 Dislike Slightly
			5 Neither Like nor Dislike
			6 Like Slightly
			7 Like Moderately
			8 Like Very Much
			9 Like Extremely
p1_q8		How much do you like bacon (pork, turkey, etc.)?	0 Never Tasted
			1 Dislike Extremely
			2 Dislike Very Much
			3 Dislike Moderately
			4 Dislike Slightly
			5 Neither Like nor Dislike
			6 Like Slightly
			7 Like Moderately
			8 Like Very Much
			9 Like Extremely
p1_q9		How much do you like canned meat or fish (i.e., chicken, tuna, salmon, etc.)?	0 Never Tasted
			1 Dislike Extremely
			2 Dislike Very Much
			3 Dislike Moderately
			4 Dislike Slightly
			5 Neither Like nor Dislike
			6 Like Slightly
			7 Like Moderately
			8 Like Very Much
			9 Like Extremely
p1_q10		How much do you like hot dogs (beef, pork, chicken, etc.)?	0 Never Tasted
			1 Dislike Extremely
			2 Dislike Very Much
			3 Dislike Moderately
			4 Dislike Slightly
			5 Neither Like nor Dislike
			6 Like Slightly
			7 Like Moderately
			8 Like Very Much
			9 Like Extremely
p1_q11		How much do you like sharp cheddar cheese?	0 Never Tasted
			1 Dislike Extremely
			2 Dislike Very Much
			3 Dislike Moderately
			4 Dislike Slightly
			5 Neither Like nor Dislike
			6 Like Slightly
			7 Like Moderately
			8 Like Very Much
			9 Like Extremely
p1_q12		How much do you like semi-hard cheese (i.e., mozzarella, pepper jack, feta, etc.)?	0 Never Tasted
			1 Dislike Extremely
			2 Dislike Very Much
			3 Dislike Moderately
			4 Dislike Slightly
			5 Neither Like nor Dislike
			6 Like Slightly
			7 Like Moderately
			8 Like Very Much
			9 Like Extremely
p1_q13		How much do you like macaroni and cheese?	0 Never Tasted
			1 Dislike Extremely
			2 Dislike Very Much
			3 Dislike Moderately
			4 Dislike Slightly
			5 Neither Like nor Dislike
			6 Like Slightly
			7 Like Moderately
			8 Like Very Much
			9 Like Extremely
p1_q14		How much do you like cheese sauce (e.g., on pasta, nachos, fries, etc.)?	0 Never Tasted
			1 Dislike Extremely
			2 Dislike Very Much
			3 Dislike Moderately
			4 Dislike Slightly
			5 Neither Like nor Dislike
			6 Like Slightly
			7 Like Moderately
			8 Like Very Much
			9 Like Extremely
p1_q15		How much do you like fried chicken?	0 Never Tasted
			1 Dislike Extremely
			2 Dislike Very Much
			3 Dislike Moderately
			4 Dislike Slightly
			5 Neither Like nor Dislike
			6 Like Slightly
			7 Like Moderately
			8 Like Very Much
			9 Like Extremely
p1_q16		How much do you like gravy?	0 Never Tasted
			1 Dislike Extremely
			2 Dislike Very Much
			3 Dislike Moderately
			4 Dislike Slightly
			5 Neither Like nor Dislike
			6 Like Slightly
			7 Like Moderately
			8 Like Very Much
			9 Like Extremely
p1_q17		How much do you like meat kebabs?	0 Never Tasted
			1 Dislike Extremely
			2 Dislike Very Much
			3 Dislike Moderately
			4 Dislike Slightly
			5 Neither Like nor Dislike
			6 Like Slightly
			7 Like Moderately
			8 Like Very Much
			9 Like Extremely
p1_q18		How much do you like hamburgers?	0 Never Tasted
			1 Dislike Extremely
			2 Dislike Very Much
			3 Dislike Moderately
			4 Dislike Slightly
			5 Neither Like nor Dislike
			6 Like Slightly
			7 Like Moderately
			8 Like Very Much
			9 Like Extremely
p1_q19		How much do you like chicken nuggets?	0 Never Tasted
			1 Dislike Extremely
			2 Dislike Very Much
			3 Dislike Moderately
			4 Dislike Slightly
			5 Neither Like nor Dislike
			6 Like Slightly
			7 Like Moderately
			8 Like Very Much
			9 Like Extremely
p1_q20		How much do you like caramelized meat drippings?	0 Never Tasted
			1 Dislike Extremely
			2 Dislike Very Much
			3 Dislike Moderately
			4 Dislike Slightly
			5 Neither Like nor Dislike
			6 Like Slightly
			7 Like Moderately
			8 Like Very Much
			9 Like Extremely
p1_q22		How much do you like dried fruit (e.g., raisins, apricots, etc.)?	0 Never Tasted
			1 Dislike Extremely
			2 Dislike Very Much
			3 Dislike Moderately
			4 Dislike Slightly
			5 Neither Like nor Dislike
			6 Like Slightly
			7 Like Moderately
			8 Like Very Much
			9 Like Extremely
p1_q21		How much do you like fruit juice?	0 Never Tasted
			1 Dislike Extremely
			2 Dislike Very Much
			3 Dislike Moderately
			4 Dislike Slightly
			5 Neither Like nor Dislike
			6 Like Slightly
			7 Like Moderately
			8 Like Very Much
			9 Like Extremely
p1_q23		How much do you like honey?	0 Never Tasted
			1 Dislike Extremely
			2 Dislike Very Much
			3 Dislike Moderately
			4 Dislike Slightly
			5 Neither Like nor Dislike
			6 Like Slightly
			7 Like Moderately
			8 Like Very Much
			9 Like Extremely
p1_q24		How much do you like fudge?	0 Never Tasted
			1 Dislike Extremely
			2 Dislike Very Much
			3 Dislike Moderately
			4 Dislike Slightly
			5 Neither Like nor Dislike
			6 Like Slightly
			7 Like Moderately
			8 Like Very Much
			9 Like Extremely
p1_q25		How much do you like candy?	0 Never Tasted
			1 Dislike Extremely
			2 Dislike Very Much
			3 Dislike Moderately
			4 Dislike Slightly
			5 Neither Like nor Dislike
			6 Like Slightly
			7 Like Moderately
			8 Like Very Much
			9 Like Extremely
p1_q26		How much do you like candy bars?	0 Never Tasted
			1 Dislike Extremely
			2 Dislike Very Much
			3 Dislike Moderately
			4 Dislike Slightly
			5 Neither Like nor Dislike
			6 Like Slightly
			7 Like Moderately
			8 Like Very Much
			9 Like Extremely
p1_q27		How much do you like coffee cake?	0 Never Tasted
			1 Dislike Extremely
			2 Dislike Very Much
			3 Dislike Moderately
			4 Dislike Slightly
			5 Neither Like nor Dislike
			6 Like Slightly
			7 Like Moderately
			8 Like Very Much
			9 Like Extremely
p1_q28		How much do you like whipped cream?	0 Never Tasted
			1 Dislike Extremely
			2 Dislike Very Much
			3 Dislike Moderately
			4 Dislike Slightly
			5 Neither Like nor Dislike
			6 Like Slightly
			7 Like Moderately
			8 Like Very Much
			9 Like Extremely
p1_q29		How much do you like pudding?	0 Never Tasted
			1 Dislike Extremely
			2 Dislike Very Much
			3 Dislike Moderately
			4 Dislike Slightly
			5 Neither Like nor Dislike
			6 Like Slightly
			7 Like Moderately
			8 Like Very Much
			9 Like Extremely
p1_q30		How much do you like caramel?	0 Never Tasted
			1 Dislike Extremely
			2 Dislike Very Much
			3 Dislike Moderately
			4 Dislike Slightly
			5 Neither Like nor Dislike
			6 Like Slightly
			7 Like Moderately
			8 Like Very Much
			9 Like Extremely
p1_q31		How much do you like cupcakes?	0 Never Tasted
			1 Dislike Extremely
			2 Dislike Very Much
			3 Dislike Moderately
			4 Dislike Slightly
			5 Neither Like nor Dislike
			6 Like Slightly
			7 Like Moderately
			8 Like Very Much
			9 Like Extremely
p1_q32		How much do you like chocolate mousse?	0 Never Tasted
			1 Dislike Extremely
			2 Dislike Very Much
			3 Dislike Moderately
			4 Dislike Slightly
			5 Neither Like nor Dislike
			6 Like Slightly
			7 Like Moderately
			8 Like Very Much
			9 Like Extremely
p1_q33		How much do you like chocolate cake?	0 Never Tasted
			1 Dislike Extremely
			2 Dislike Very Much
			3 Dislike Moderately
			4 Dislike Slightly
			5 Neither Like nor Dislike
			6 Like Slightly
			7 Like Moderately
			8 Like Very Much
			9 Like Extremely
p1_q34		How much do you like brownies?	0 Never Tasted
			1 Dislike Extremely
			2 Dislike Very Much
			3 Dislike Moderately
			4 Dislike Slightly
			5 Neither Like nor Dislike
			6 Like Slightly
			7 Like Moderately
			8 Like Very Much
			9 Like Extremely
p1_q35		How much do you like cheesecake?	0 Never Tasted
			1 Dislike Extremely
			2 Dislike Very Much
			3 Dislike Moderately
			4 Dislike Slightly
			5 Neither Like nor Dislike
			6 Like Slightly
			7 Like Moderately
			8 Like Very Much
			9 Like Extremely
p1_q36		How much do you like vanilla wafers?	0 Never Tasted
			1 Dislike Extremely
			2 Dislike Very Much
			3 Dislike Moderately
			4 Dislike Slightly
			5 Neither Like nor Dislike
			6 Like Slightly
			7 Like Moderately
			8 Like Very Much
			9 Like Extremely
p1_q37		How much do you like sugar cookies?	0 Never Tasted
			1 Dislike Extremely
			2 Dislike Very Much
			3 Dislike Moderately
			4 Dislike Slightly
			5 Neither Like nor Dislike
			6 Like Slightly
			7 Like Moderately
			8 Like Very Much
			9 Like Extremely
p1_q38		How much do you like croissants?	0 Never Tasted
			1 Dislike Extremely
			2 Dislike Very Much
			3 Dislike Moderately
			4 Dislike Slightly
			5 Neither Like nor Dislike
			6 Like Slightly
			7 Like Moderately
			8 Like Very Much
			9 Like Extremely
p1_q39		How much do you like chocolate chip cookies?	0 Never Tasted
			1 Dislike Extremely
			2 Dislike Very Much
			3 Dislike Moderately
			4 Dislike Slightly
			5 Neither Like nor Dislike
			6 Like Slightly
			7 Like Moderately
			8 Like Very Much
			9 Like Extremely
p1_q40		How much do you like apple pie?	0 Never Tasted
			1 Dislike Extremely
			2 Dislike Very Much
			3 Dislike Moderately
			4 Dislike Slightly
			5 Neither Like nor Dislike
			6 Like Slightly
			7 Like Moderately
			8 Like Very Much
			9 Like Extremely
p1_q41		How much do you like doughnuts?	0 Never Tasted
			1 Dislike Extremely
			2 Dislike Very Much
			3 Dislike Moderately
			4 Dislike Slightly
			5 Neither Like nor Dislike
			6 Like Slightly
			7 Like Moderately
			8 Like Very Much
			9 Like Extremely
Part 2 Instructions The objective is to determine how you prefer your food (which is not necessarily the way you usually eat your food). The items listed make reference to foods which have not yet been seasoned. Before you begin, please review the following example. How do you prefer to eat steak?			
P2 Example2		Check the box that corresponds to the way you prefer to eat steak. Each number corresponds to a steak with a quantity of butter as shown in the photo (0, 2, 4) or an intermediate amount to that (1, 3). Number 5 corresponds to a steak with a quantity of butter even more than that in number 4. To respond, choose the number representing how much butter you prefer to eat on your steak. If you love to eat your steak with a big knob of butter, but for reasons of health you generally consume it without butter then answer how you would like and not what you usually eat. If you do not like steak, then check the box "Dislike Steak".	0 (none), 1, 2, 3, 4, 5 (A Lot), 6 (Dislike Steak)
p2_q1		How do you prefer boiled eggs?	
p2_q1a		Check the box that corresponds to how much salt you prefer to eat on boiled eggs.	0 (None), 1, 2, 3, 4, 5 (A Lot), 6 (Dislike Boiled Eggs)
p2_q2		How do you prefer corn on the cob?	
p2_q2a		Check the box that corresponds to how much butter you prefer to eat on corn on the cob.	0 (None), 1, 2, 3, 4, 5 (A Lot), 6 (Dislike Corn on the Cob)
p2_q3		How do you prefer deli meat sandwiches?	
p2_q3a		Check the box that corresponds to how much mayonnaise you prefer to eat on deli meat sandwiches.	0 (None), 1, 2, 3, 4, 5 (A Lot), 6 (Dislike Deli Meat Sandwiches)
p2_q4		How do you prefer green beans?	
p2_q4a		Check the box that corresponds to how much butter you prefer to eat on green beans.	0 (None), 1, 2, 3, 4, 5 (A Lot), 6 (Dislike Green Bean)
p2_q5		How do you prefer green beans?	
p2_q5a		Check the box that corresponds to how much salt you prefer to eat on green beans.	0 (None), 1, 2, 3, 4, 5 (A Lot), 6 (Dislike Green Bean)
p2_q6		How do you prefer mashed potatoes?	
p2_q6a		Check the box that corresponds to how much salt you prefer to eat on mashed potatoes.	0 (None), 1, 2, 3, 4, 5 (A Lot), 6 (Dislike Mashed Potatoes)
p2_q7		How do you prefer steaks?	
p2_q7a		Check the box that corresponds to how much butter you prefer to eat on steaks.	0 (None), 1, 2, 3, 4, 5 (A Lot), 6 (Dislike Steaks)
p2_q8		How do you prefer chicken breasts?	
p2_q8a		Check the box that corresponds to how much salt you prefer to eat on chicken breasts.	0 (None), 1, 2, 3, 4, 5 (A Lot), 6 (Dislike Chicken Breasts)
p2_q9		How do you prefer salmon?	
p2_q9a		Check the box that corresponds to how much dill sauce you prefer to eat on salmon.	0 (None), 1, 2, 3, 4, 5 (A Lot), 6 (Dislike Salmon)
p2_q10		How do you prefer cheese sandwiches?	
p2_q10a		Check the box that corresponds to how much mayonnaise you prefer to eat on cheese sandwiches.	0 (None), 1, 2, 3, 4, 5 (A Lot), 6 (Dislike Cheese Sandwiches)
p2_q11		How do you prefer toast?	
p2_q11a		Check the box that corresponds to how much butter you prefer to eat on toast.	0 (None), 1, 2, 3, 4, 5 (A Lot), 6 (Dislike Toast)
p2_q12		How do you prefer toast?	
p2_q12a		Check the box that corresponds to how much jam you prefer to eat on toast.	0 (None), 1, 2, 3, 4, 5 (A Lot), 6 (Dislike Toast)
p2_q13		How do you prefer toast?	
p2_q13a		Check the box that corresponds to how much nutella you prefer to eat on toast.	0 (None), 1, 2, 3, 4, 5 (A Lot), 6 (Dislike Toast)
p2_q14		How do you prefer strawberries?	
p2_q14a		Check the box that corresponds to how much sugar you prefer to eat on strawberries.	0 (None), 1, 2, 3, 4, 5 (A Lot), 6 (Dislike Strawberries)
p2_q15		How do you prefer yogurt?	
p2_q15a		Check the box that corresponds to how much fruit you prefer to eat on yogurt.	0 (None), 1, 2, 3, 4, 5 (A Lot), 6 (Dislike Yogurt)
p2_q16		How do you prefer pancakes?	
p2_q16a		Check the box that corresponds to how much syrup you prefer to eat on pancakes.	0 (None), 1, 2, 3, 4, 5 (A Lot), 6 (Dislike Pancakes)
p2_q17		How do you prefer pancakes?	
p2_q17a		Check the box that corresponds to how much butter you prefer to eat on pancakes.	0 (None), 1, 2, 3, 4, 5 (A Lot), 6 (Dislike Pancakes)
p2_q18		How do you prefer brownies?	
p2_q18a		Check the box that corresponds to how much whipped cream you prefer to eat on brownies.	0 (None), 1, 2, 3, 4, 5 (A Lot), 6 (Dislike Brownies)
p2_q19		How do you prefer ice cream?	
p2_q19a		Check the box that corresponds to how much whipped cream you prefer to eat on ice cream.	0 (None), 1, 2, 3, 4, 5 (A Lot), 6 (Dislike Ice Cream)
Part 3 Instructions The objective is to determine what you prefer to drink. A new restaurant will open soon at a location near you. The restaurant wants to offer customers' favorite drinks on the menu. They have created the following survey to determine drink preferences. In the list of drinks below, select up to 3 drinks that you prefer.			
p3_q1		In the list of drink below, select the drinks that you prefer. You can select up to 3 choices. If you do not like any of the drink choices select "None".	0 Fruit Juice (apricot, pear, grape, apple, orange, etc.)
			1Tomato Juice
			2 Soda (lemonade, cola, diet cola, orange soda, etc.)
			3 Flavored Water
			4 Sparkling Water (with or without lemon)
			5 Mineral Water (with or without lemon)
			6 None
Part 4 Instructions The objective is to determine your food consumption behavior. Please answer spontaneously. There are no right or wrong answers.			
p4_q1		You are given bread at a party. You realize there is no butter. Does it bother you to eat bread without butter?	0 (Not At All), 1, 2, 3, 4, 5, 6, 7, 8, 9 (Very Much), 10 (Dislike Bread)
p4_q2		You are in a restaurant and order yogurt with fruit. After tasting it you realize that it is not very sweet. Does it bother you to eat yogurt that is not very sweet?	0 (Not At All), 1, 2, 3, 4, 5, 6, 7, 8, 9 (Very Much), 10 (Dislike Yogurt)
p4_q3		You are in a cafe and the waiter brings your favorite hot drink (coffee, tea, herbal tea). There is no sugar on the table. Does it bother you to drink your hot drink without sugar?	0 (Not At All), 1, 2, 3, 4, 5, 6, 7, 8, 9 (Very Much), 10 (Dislike Hot Drinks)
p4_q5		Do you add salt to your meal before tasting it?	0 Never
			1 Rarely
			2 Sometimes
			3 Often
			4 Always
p4_q6		You go on a BBQ with friends. You bring bread, egg salad, and tomatoes. Do you remember to bring salt?	0 Never
			1 Rarely
			2 Sometimes
			3 Often
			4 Always
p4_q7		You are eating a cup of ice cream. Do you put whipped cream on top?	0 Never
			1 Rarely
			2 Sometimes
			3 Often
			4 Always
p4_q9		Do you eat nutella or chocolate spread with a spoon?	0 Never
			1 Rarely
			2 Sometimes
			3 Often
			4 Always
p4_q10		Do you eat jam with a spoon?	0 Never
			1 Rarely
			2 Sometimes
			3 Often
			4 Always
p4_q11		Do you add salt to the water used to cook pasta?	0 Never
			1 Rarely
			2 Sometimes
			3 Often
			4 Always
p4_q12		How do you prefer your soup?	0 Not Salty
			1 Slightly Salty
			2 Moderately Salty
			3 Rather Salty
			4 Very Salty
			5 Dislike Soup
p4_q13		How do you prefer your soup?	0 Without Cream
			1 Small Amount of Cream
			2 Moderate Amount of Cream
			3 Large Amount of Cream
			4 Very Large Amount of Cream
			5 Dislike Soup
p4_q14		How do you prefer your pasta?	0 Not Salty
			1 Slightly Salty
			2 Moderately Salty
			3 Rather Salty 4 Very Salty 5 Dislike Pasta
			4 Very Salty
			5 Dislike Pasta
p4_q15		How do you prefer your pasta?	0 Without Butter
			1 Small Amount of Butter
			2 Moderate Amount of Butter
			3 Large Amount of Butter
			4 Very Large Amount of Butter
			5 Dislike Pasta
p4_q16		How do you prefer your mashed potatoes?	0 Without Butter
			1 Small Amount of Butter
			2 Moderate Amount of Butter
			3 Large Amount of Butter
			4 Very Large Amount of Butter
			5 Dislike Mashed Potatoes
p4_q17		How do you prefer your fries?	0 Not Salty
			1 Slightly Salty
			2 Moderately Salty
			3 Rather Salty
			4 Very Salty
			5 Dislike Fries
p4_q18		How do you prefer your steak?	0 Not Salty
			1 Slightly Salty
			2 Moderately Salty
			3 Rather Salty
			4 Very Salty
			5 Dislike Steak
p4_q19		How do you prefer your yogurt?	0 Not Sweet
			1 Slightly Sweet
			2 Moderately Sweet
			3 Rather Sweet
			4 Very Sweet
			5 Dislike Yogurt
p4_q20		How do you prefer your strawberries?	0 Without Cream
			1 Small Amount of Cream
			2 Moderate Amount of Cream
			3 Large Amount of Cream
			4 Very Large Amount of Cream
			5 Dislike Strawberries
p4_q21		How do you prefer your pancakes?	0 Without Syrup
			1 Small Amount of Syrup
			2 Moderate Amount of Syrup
			3 Large Amount of Syrup
			4 Very Large Amount of Syrup
			5 Dislike Pancakes
p4_q22		How do you prefer your pancakes?	0 Without Nutella
			1 Small Amount of Nutella
			2 Moderate Amount of Nutella
			3 Large Amount of Nutella
			4 Very Large Amount of Nutella
			5 Dislike Pancakes
p4_q23		How do you prefer your tea?	0 Not Sweet
			1 Slightly Sweet
			2 Moderately Sweet
			3 Rather Sweet
			4 Very Sweet
			5 Dislike Tea
p4_q24		How do you prefer your coffee?	0 Not Sweet
			1 Slightly Sweet
			2 Moderately Sweet
			3 Rather Sweet
			4 Very Sweet
			5 Dislike Coffee

**Table 6:** Baseline Clinical Characteristics Values presented are mean (standard deviation). FD5, fasting day 5; RD3, refeed day 3; BMI, body mass index; SBP, systolic blood pressure; DBP, diastolic blood pressure; cm, centimeter; kg, kilogram; m2, square meter; mm, millimeter; Hg, mercury; y, years.

	N=30 Enrolled at Baseline	n=25 Remaining at FD5	n=23 Remaining at RD3
Age (y)	47.4 (13.0)	47.2 (12.2)	47.7 (10.7)
Weight (kg)	84.5 (22.6)	81.4 (18.8)	79.8 (15.3)
BMI (kg/m^2^)	29.3 (8.1)	28.0 (6.1)	27.5 (5.2)
SBP (mm/Hg)	116.2 (14.3)	113.9 (13.6)	113.8 (13.8)
DBP (mm/Hg)	73.9 (8.4)	73.1 (8.7)	73.6 (8.9)
